# Prevalence and Risk Factors for Cerebral Microbleeds in Chinese Patients With Acute Ischemic Stroke

**DOI:** 10.1002/brb3.71495

**Published:** 2026-05-27

**Authors:** Ruiming Zhu, Yinglin Yang, Xiaoyan Chen, Lei Hou, Xiangshu Cheng, Mianwang He

**Affiliations:** ^1^ Department of Neurology, Translational Medicine Center Huaihe Hospital of Henan University Kaifeng Henan China; ^2^ Department of Neurology, Chinese PLA General Hospital Chinese PLA Medical School Beijing China; ^3^ Department of Pharmacy, Medical Supplies Center of Chinese PLA General Hospital Chinese PLA Medical School Beijing China; ^4^ Institute for Brain Sciences Research Henan University Kaifeng Henan China

**Keywords:** acute ischemic stroke, cerebral microbleeds, hypertension, susceptibility‐weighted imaging, white matter hyperintensities

## Abstract

**Background:**

Cerebral microbleeds (CMBs) are recognized as significant markers in acute ischemic stroke (AIS). Although prior studies conducted in southern China have reported CMB prevalence ranging from 27.8% to 56.7% among patients with AIS, data from northern China remain limited, and associated risk factors are not well defined. The aim of this study was to assess the prevalence and determinants of CMBs in a cohort of Chinese patients with AIS.

**Methods:**

In this hospital‐based retrospective study, 171 consecutive patients diagnosed with AIS were assessed for the presence, number, and anatomical distribution of CMBs using standardized 3.0 Tesla susceptibility‐weighted magnetic resonance imaging protocols. Baseline demographic and clinical variables were collected and analyzed in relation to CMB presence, burden, and location.

**Results:**

The cohort (mean age 64.8 ± 13.0 years; 73.7% male) demonstrated a CMB prevalence of 49.1%, with variation across TOAST subtypes: large artery atherosclerosis (LAA) (59.4%), small vessel disease (SVD) (47.5%), cardioembolism (21.4%), and other etiologies (25.0%). Multivariate logistic regression identified hypertension (adjusted odds ratio [AOR] = 2.07, 95% confidence interval [CI]: 1.01–4.24) and severe white matter hyperintensity (WMH) (AOR = 4.38, 95% CI: 1.69–11.35) as independent risk factors for the presence of CMBs. Patients with ≥ 3 CMBs had a significantly higher proportion of SVD subtype (58.1% vs. 31.7%, *p* < 0.01) and lower levels of low‐density lipoprotein cholesterol (1.85 vs. 2.38 mmol/L, *p* < 0.01) when compared with those with 1–2 CMBs. Deep and infratentorial CMBs were associated with a higher frequency of SVD (59.3% and 51.4% vs. 15.0%, *p* < 0.01) and moderate‐to‐severe WMH (51.8% and 81.0% vs. 45.0%, *p* < 0.05) when compared with strictly lobar CMBs.

**Conclusion:**

Approximately half of the patients with AIS exhibited CMBs, particularly those classified under LAA and SVD subtypes. Hypertension and severe WMH were independently associated with CMB presence. Distinct clinical profiles were observed based on CMB burden and location. Further research is warranted to elucidate underlying mechanisms and clinical implications.

## Graphical abstract

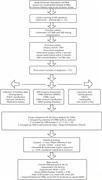

**Abbreviations**
AIS: acute ischemic stroke
CAA: cerebral amyloid angiopathy
Ccr: creatinine clearance rate
CE: cardioembolism
Chinese PLA General Hospital: Chinese People's Liberation Army General Hospital
CMBs: cerebral microbleeds
CSVD: cerebral small vessel disease
DM: diabetes mellitus
FBG: fasting blood glucose
HbA1c: glycated hemoglobin
HCY: homocysteine
HDL: high density lipoprotein
HDL‐cholesterol: high‐density lipoprotein cholesterol
HTN: hypertension
IQR: interquartile range
LAA: large artery atherosclerosis
LDL: low density lipoprotein
LDL‐cholesterol: low‐density lipoprotein cholesterol
MARS: Microbleed Anatomical Rating Scale
MRI: magnetic resonance imaging
ODC: other determined causes
Scr: serum creatinine
SPSS: Statistical Package for the Social Sciences
SVD: small vessel disease
SWI: susceptibility‐weighted images
SWI‐MRI: susceptibility‐weighted magnetic resonance imaging
T2*GRE: T2*‐weighted gradient‐recalled echo
TC: total cholesterol
TG: triglycerides
UA: uric acid
WMH: white matter hyperintensity

## 1 Introduction

Cerebral microbleeds (CMBs) are characterized as small, round, or ovoid, homogeneous hypointense lesions observed on gradient‐echo T2*‐weighted gradient‐recalled echo (T2*‐GRE) or susceptibility‐weighted imaging (SWI) sequences of magnetic resonance imaging (MRI) (Greenberg et al. 2009). The presence of CMBs has been significantly associated with an elevated risk of intracranial hemorrhagic transformation, hemorrhage, and recurrent stroke in patients diagnosed with acute ischemic stroke (AIS), highlighting their clinical relevance in stroke management (Nagaraja et al. 2018; Dar et al. 2018; Charidimou et al. 2018). Five independent studies conducted in southern China have reported a wide range of CMB prevalence among patients with AIS, with rates varying from 27.8%, 35.5%, 42.6%, and 49.69% to 56.7% (Soo et al. 2008; Fan et al. 2003; Zhou et al. 2010; Lei et al. 2018; Meng et al. 2020). These values were consistently higher when compared with those documented in other populations, including Thai (19.5%), Egyptian (26.3%), and British (23%) cohorts (Potigumjon et al. 2017; Nasreldein et al. 2025; Werring et al. 2005). However, data pertaining to the prevalence of CMBs in patients with AIS from northern China remain limited. This gap underscores the need for additional region‐specific studies to facilitate a comprehensive understanding of the national prevalence of CMBs in China.

In addition, the underlying risk factors contributing to the development of CMBs have not been fully elucidated, emphasizing the necessity for further investigation. Therefore, the aim of this study was to assess the prevalence and identify factors associated with CMBs in Chinese patients with AIS.

## 2 Materials and Methods

### 2.1 Patients and Evaluation

Ethical approval for the study was obtained from the Ethics Review Board of the Chinese People's Liberation Army General Hospital (Chinese PLA General Hospital). A retrospective analysis was conducted involving consecutive patients diagnosed with AIS who were admitted to the Department of Neurology, First Medical Center, Chinese PLA General Hospital, between January 1, 2023 and June 30, 2023.

Inclusion was limited to patients who underwent SWI using a 3.0‐Tesla MRI scanner during hospitalization. Patients were excluded if they had (1) a history of intracerebral or subarachnoid hemorrhage, (2) an intracranial neoplasm, (3) recent intracranial surgery (within the past 3 months), (4) significant head trauma within the preceding 3 months, or (5) contraindications to MRI or unavailable SWI data.

### 2.2 Data Collection

Comprehensive baseline demographic and clinical characteristics were obtained, including (1) demographic variables: age and sex; (2) clinical assessments: Trial of Org 10172 in Acute Stroke Treatment (TOAST) stroke classification; (3) treatment history: prehospital antithrombotic therapy (including antiplatelet agents) and thrombolytic treatment (intravenous thrombolysis); and (4) vascular risk factors: hypertension (HTN), diabetes mellitus (DM), coronary heart disease, current smoking status, and alcohol consumption.

HTN was defined based on established criteria: either current use of antihypertensive medication or documentation of elevated blood pressure (systolic ≥ 140 mm Hg and/or diastolic ≥ 90 mm Hg) on at least two separate measurements obtained after the acute stroke phase.

Laboratory investigations included the following parameters—(1) markers of glucose metabolism: fasting plasma glucose and glycated hemoglobin (HbA1C); (2) lipid profile: total cholesterol (TC), low‐density lipoprotein (LDL) cholesterol, high‐density lipoprotein cholesterol (HDL‐cholesterol), and triglycerides (TG); and (3) additional metabolic indicators including serum uric acid (UA), creatinine, and homocysteine (HCY) levels. All biochemical analyses were conducted in the central laboratory of the Chinese People's Liberation Army General Hospital, in accordance with standardized protocols.

### 2.3 CMBs

The presence and characteristics of CMBs were assessed using the Microbleed Anatomical Rating Scale (MARS) (Gregoire et al. 2009). CMBs were classified according to anatomical location into three categories—(1) strictly lobar: restricted to the cerebral cortex, subcortical junction, or insula; (2) strictly deep/infratentorial: located within deep structures (basal ganglia, thalamus, corona radiata, internal/external capsule) or infratentorial regions (brainstem, cerebellum); and (3) mixed: present in both lobar and deep/infratentorial locations. The burden of CMBs was stratified based on quantity as follows: mild (1–2 microbleeds), moderate (3–10 microbleeds), and severe (> 10 microbleeds). SWI scans were independently assessed by two board‐certified neurologists blinded to all clinical data. Any discrepancies in interpretation were resolved through consultation with a neuroradiologist, and final determinations were reached by consensus.

### 2.4 White Matter Hyperintensity (WMH) Grading

The severity of WMH was assessed using the Fazekas scale, a visual rating method established by the European Task Force on Age‐Related White Matter Changes. WMH severity was graded on a four‐point scale: 0 (no lesions), 1 (mild focal lesions), 2 (moderate, beginning confluent lesions), and 3 (severe diffuse involvement of entire regions of white matter) (Wahlund et al. 2001).

### 2.5 Statistical Analysis

All statistical analyses were performed using the Statistical Package for the Social Sciences (SPSS), version 26.0 (SPSS Inc., Chicago, Illinois, USA). Continuous variables were presented as mean ± standard deviation, while categorical variables were expressed as numbers and percentages (*n* [%]). The Student's *t*‐test was applied to compare continuous variables, and the chi‐squared (*χ* ^2^) test or Fisher's exact test was used for categorical comparisons, as appropriate. For non‐normally distributed variables, data were reported as medians with interquartile range (IQR), and comparisons between two groups were conducted using the Mann–Whitney *U* test; the Kruskal–Wallis *H* test was used for comparisons involving more than two groups. Logistic regression analysis was applied to identify factors associated with the presence of CMBs. A *p* < 0.05 was considered statistically significant.

## 3 Results

### 3.1 Demographics

A total of 372 patients diagnosed with AIS were initially reviewed. Following the application of inclusion and exclusion criteria, 201 cases (54.0%) were excluded, resulting in a final study cohort comprising 171 patients. Note that 22 patients (12.9%) were aged ≤ 50 years, 138 patients (80.7%) were aged 50–85 years, and 11 patients (6.4%) were aged > 85 years. The mean age of the cohort was 64.8 ± 13.0 years, with a predominance of male patients (73.7%, *n* = 126). HTN was observed in 67.3% of patients, followed by a history of smoking (44.4%), alcohol consumption (43.3%), and DM (34.5%).

### 3.2 Stroke Subtypes and Imaging Characteristics

The distribution of stroke subtypes, classified according to the TOAST criteria, was as follows: large artery atherosclerosis (LAA) in 40.4% (*n* = 69), small vessel disease (SVD) in 46.8% (*n* = 80), cardioembolism (CE) in 8.2% (*n* = 14), and other determined causes (ODC) in 4.6% (*n* = 8).

WMH severity, graded using the Fazekas scale, demonstrated the following distribution: Grade 0 in 2.3% (*n* = 4), Grade 1 in 47.4% (*n* = 81), Grade 2 in 28.1% (*n* = 48), and Grade 3 in 22.2% (*n* = 38) (Table 1).

**TABLE 1:** Baseline demographic characteristics of the study cohort.

Category	*N* = 171
**Age, mean ± SD, (yrs)**	64.8 ± 13.0
**Age group, *n* (%)**	
≤ 50 (years)	22 (12.9%)
> 50 (years)	149 (87.1%)
**Sex, *n* (%)**	
Male	126 (73.7%)
Female	45 (26.3%)
**Stroke etiology, *n* (%)**	
LAA	69 (40.4%)
SVD	80 (46.8%)
CE	14 (8.2%)
ODC	8 (4.6%)
UND	0 (0)
**Risk factors, *n* (%)**	
HTN	115 (67.3%)
DM	59 (34.5%)
Smoking	76 (44.4%)
Drinking	74 (43.3%)
**Fazekas score, *n* (%)**	
Grade 0	4 (2.3%)
Grade 1	81 (47.4%)
Grade 2	48 (28.1%)
Grade 3	38 (22.2%)
**CMBs number,*n* (%)**	
1–2	41 (48.8%)
3–10	32 (38.1%)
> 10	11 (13.1%)
**Location of CMBs, *n* (%)**	
Lobar	20 (23.8%)
Deep or infratentorial	27 (32.1%)
Mixed	37 (44.1%)
**CMBs, *n* (%)**	
Patients with CMBs	84 (49.1%)
Patients without CMBs	87 (50.9%)

Abbreviations: CE, cardioembolism; CMBs, cerebral microbleeds; DM, diabetes mellitus; HTN, hypertension; LAA, large artery atherosclerosis; ODC, other determined causes; SVD, small vessel disease; UND, stroke of undetermined etiology.

### 3.3 Prevalence and Risk Factors of CMBs

CMBs were identified in 49.1% (84/171) of the study cohort (Table 1). The prevalence of CMBs varied across stroke subtypes as defined by the TOAST classification: 59.4% (41/69) in patients with LAA, 47.5% (38/80) in those with SVD, 21.4% (3/14) in CE, and 25.0% (2/8) in patients with strokes of other determined etiologies.

Patients with CMBs demonstrated a higher mean age compared with those without CMBs (65.4 ± 12.2 vs. 64.2 ± 13.9 years); however, this difference was not statistically significant (*p* = 0.56). Lobar CMBs tended to be more common among older patients (68.6 ± 14.7 years), compared with those with deep/infratentorial (65.7 ± 10.8 years) or mixed CMBs (63.7 ± 10.9 years), although these differences did not reach statistical significance (*p* = 0.33).

### 3.4 CMBs Burden and Distribution Patterns

Among patients with CMBs (*n* = 84), 48.8% (*n* = 41) had 1–2 microbleeds, 38.1% (*n* = 32) had 3–10 microbleeds, and 13.1% (*n* = 11) had more than 10 microbleeds. Among patients with CMBs > 10, 7 were classified as LAA, 2 as SVD, 1 as CE, and 1 as ODC. Compared with the group of patients with ≤ 2 CMBs, a higher proportion of those with ≥ 3 CMBs experienced strokes attributed to SVD (58.1% vs. 31.7%, *p* < 0.01) and exhibited Fazekas Grade 3 WMH (46.5% vs. 19.5%, *p* < 0.05). In addition, mean LDL cholesterol levels were significantly lower in patients with ≥ 3 CMBs compared to those with ≤ 2 CMBs (1.85 [1.38, 2.77] mmol/L vs. 2.38 [1.92, 3.03] mmol/L, *p* < 0.01) (Table 2).

**TABLE 2:** Factors associated with the presence of CMBs.

Terms	Without CMBs (*n* = 87)	CMBs (*n* = 84)	*p* value	CMBs *n* ≤ 2 (*n* = 41)	CMBs *n* ≥ 3 (*n* = 43)	Statistical method	*p* value
**Sex, *n* (%)**						*χ* ^2^	
Female	24 (27.6)	21 (25.0)	0.70	8 (19.5)	13 (30.2)		0.26
**Age (years)**	64.2 ± 13.9	65.4 ± 12.2	0.56	65.4 ± 12.3	65.3 ± 12.2	ANOVA	0.95
**Smoking, *n* (%)**						*χ* ^2^	
Yes	42 (48.3)	34 (40.5)	0.30	19 (46.3)	15 (34.9)		0.28
**Drinking, *n* (%)**						*χ* ^2^	
Yes	37 (42.5)	37 (44.0)	0.84	20 (48.8)	17 (39.5)		0.39
**DM, *n* (%)**						*χ* ^2^	
Yes	30 (34.5)	29 (34.5)	0.99	15 (36.6)	14 (32.6)		0.70
**HTN, *n* (%)**						*χ* ^2^	
Yes	52 (59.8)	63 (75.0)	0.034*	29 (70.7)	34 (79.1)		0.38
**History of intravenous thrombolysis, *n* (%)**						*χ* ^2^	
Yes	20 (23.0)	18 (21.7)	0.84	7 (17.5)	11 (25.6)		0.37
**Use of antiplatelet drugs, *n* (%)**						*χ* ^2^	
A	13 (14.9)	15 (17.9)	0.87	6 (14.6)	9 (20.9)		0.51
A + C	6 (6.9)	6 (7.1)		2 (4.9)	4 (9.3)		
**Use of anticoagulants, *n* (%)**						Fisher	
Yes	4 (4.6)	2 (2.4)	0.68	1 (2.4)	1 (2.3)		1.00
**Biochemical indicators**							
HbA1c (mmol/L)	6.10 (5.65, 7.20)	6.10 (5.50, 7.05)	0.67	6.10 (5.5, 7.3)	6.00 (5.6, 6.50)	Kruskal–Wallis	0.71
FBG (mmol/L)	5.32 (4.69, 6.33)	5.41 (4.51, 6.68)	0.41	5.70 (4.87, 7.32)	5.28 (4.42, 6.40)	Kruskal–Wallis	0.43
TG (mmol/L)	1.33 (0.94, 1.68)	1.29 (1.05, 1.95)	0.52	1.22 (0.99, 1.72)	1.38 (1.06, 2.10)	Kruskal–Wallis	0.32
TC (mmol/L)	4.08 (3.28, 4.63)	3.70 (3.20, 4.56)	0.24	3.84 (3.48, 4.50)	3.59 (2.94, 4.56)	Kruskal–Wallis	0.12
LDL (mmol/L)	2.47 ± 0.85	2.30 ± 0.86	0.19	2.38 (1.92, 3.03)	1.85 (1.38, 2.77)	ANOVA	0.001*
HDL (mmol/L)	1.06 (0.89, 1.21)	1.08 (0.79, 1.27)	0.60	1.07 ± 0.28	1.07 (0.79, 1.22)	Kruskal–Wallis	0.66
UA (µmol/L)	305.70 (241.75, 392.90)	307.85 (253.4, 382.70)	0.87	320.66 ± 78.84	286.6 (243.1, 407.3)	Kruskal–Wallis	0.48
Ccr (mL/min)	88.70 (73.90, 95.50)	83.00 (65.20, 92.50)	0.07	85.80 (63.5, 93.3)	82.00 (68.3, 92.5)	Kruskal–Wallis	0.80
HCY (µmol/L)	13.15 (10.35, 17.95)	14.75 (11.20, 18.50)	0.12	17.40 (11.9, 19.8)	13.60 (10.9, 18.1)	Kruskal–Wallis	0.11
**Stroke etiology, *n* (%)**						Fisher	
LAA	28 (32.2)	41(48.8)	0.028*	27 (65.9)	14 (32.6)		0.004*
SVD	42 (48.3)	38 (45.2)		13 (31.7)	25 (58.1)		
CE	11 (12.6)	3 (3.6)		0 (0.0)	3 (7.0)		
ODC	6 (6.9)	2 (2.4)		1 (2.4)	1 (2.3)		
UND	0 (0.0)	0 (0.0)		0 (0.0)	0 (0.0)		
**Fazekas score, *n* (%)**						Fisher	
Grade 1	49 (56.3)	32 (38.1)	0.001*	20 (48.8)	12 (27.9)		0.026*
Grade 2	24 (27.6)	24 (28.6)		13 (31.7)	11 (25.6)		
Grade 3	10 (11.5)	28 (33.3)		8 (19.5)	20 (46.5)		

*Note*: Male = (140 − age)*weight (kg)/72*Scr, female = (140 − age)*weight (kg)*0.85/72*Scr.

Abbreviations: A, aspirin; C, clopidogrel; Ccr, creatinine clearance rate; FBG, fasting blood glucose; HbA1c, glycated hemoglobin; HCY, homocysteine; HDL, high density lipoprotein; LDL, low density lipoprotein; Scr, serum creatinine; TC, total cholesterol; TG, triglycerides; UA, uric acid.

*p < 0.05.

Regarding anatomical distribution, CMBs were classified as strictly lobar in 23.8% (*n* = 20), strictly deep/infratentorial in 32.1% (*n* = 27), and mixed in 44.1% (*n* = 37). Compared with patients in the strictly lobar CMB group, those with deep/infratentorial or mixed CMBs more frequently had strokes related to SVD (59.3% and 51.4% vs. 15.0%, *p* < 0.01) and demonstrated a higher prevalence of moderate‐to‐severe WMH (51.8% and 81.0% vs. 45.0%, *p* < 0.05) (Table 3).

**TABLE 3:** Factors associated with the location of CMBs.

Terms	Lobar (*n* = 20)	Deep or infratentorial (*n* = 27)	Mixed (*n* = 37)	Statistical method	*p* value
**Sex, *n* (%)**				*χ* ^2^	
Female	6 (30.0)	7 (25.9)	8 (21.6)		0.78
**Age (years)**	68.6 ± 14.7	65.7 ± 10.8	63.7 ± 10.9	ANOVA	0.33
**Smoking, *n* (%)**				*χ* ^2^	
Yes	6 (30.0)	13 (48.1)	15 (40.5)		0.46
**Drinking, *n* (%)**				*χ* ^2^	
Yes	8 (40.0)	10 (37.0)	20 (54.1)		0.35
**DM, *n* (%)**				*χ* ^2^	
Yes	8 (40.0)	11 (40.7)	11 (29.7)		0.60
**HTN, *n* (%)**				*χ* ^2^	
Yes	15 (75.0)	18 (66.7)	29 (78.4)		0.57
**History of intravenous thrombolysis, *n* (%)**				Fisher	
Yes	5 (25.0)	8 (29.6)	6 (16.2)		0.52
**Use of antiplatelet drugs, *n* (%)**				Fisher	
A	4 (20)	5 (18.5)	6 (16.2)		0.89
A + C	1 (5)	1 (3.7)	4 (10.8)		
**Use of anticoagulants, *n* (%)**				Fisher	
Yes	0 (0.0)	0 (0.0)	2 (5.4)		0.50
**Biochemical indicators**					
HbA1c (%)	6.20 (5.5, 7.3)	5.90 (5.6, 6.5)	6.2 (5.5, 7.6)	Kruskal–Wallis	0.78
FBG (mmol/L)	5.45 (4.45, 6.30)	5.70 (4.47, 7.36)	5.39 (4.71, 6.40)	Kruskal–Wallis	0.69
TG (mmol/L)	1.22 (1.08, 1.62)	1.20 (0.88, 1.73)	1.40 (1.02, 2.10)	Kruskal–Wallis	0.64
TC (mmol/L)	3.52 (3.00, 4.05)	3.85 (3.59, 4.67)	3.64 (3.03, 4.48)	Kruskal–Wallis	0.09
LDL (mmol/L)	2.19 ± 1.07	2.42 ± 0.85	2.16 ± 0.83	ANOVA	0.49
HDL (mmol/L)	0.99 (0.87, 1.06)	1.13 (0.70, 1.31)	1.12 (0.91, 1.28)	Kruskal–Wallis	0.42
UA (µmol/L)	298.25 (253.40, 382.70)	309.50 (256.40, 386.90)	309.70 (247.30, 379.30)	Kruskal–Wallis	0.99
Ccr (mL/min)	74.65 (60.00, 91.05)	83.65 (68.30, 93.60)	85.90 (71.10, 91.90)	Kruskal–Wallis	0.40
HCY (µmol/L)	15.65 (12.25, 20.00)	14.75 (11.20, 19.80)	14.65 (10.90, 18.45)	Kruskal–Wallis	0.93
**Stroke etiology, *n* (%)**				Fisher	
LAA	16 (80.0)	11 (40.7)	14 (37.8)		0.007*
SVD	3 (15.0)	16 (59.3)	19 (51.4)		
CE	1 (5.0)	0 (0.0)	2 (5.4)		
ODC	0 (0.0)	0 (0.0)	2 (5.4)		
UND	0 (0.0)	0 (0.0)	0 (0.0)		
**Fazekas score, *n* (%)**				*χ* ^2^	
Grade 1	11 (55.0)	13 (48.1)	7 (18.9)		0.048*
Grade 2	4 (20.0)	7 (25.9)	13 (35.1)		
Grade 3	5 (25.0)	7 (25.9)	17 (45.9)		

*Note*: Male = (140 − age)*weight (kg)/72*Scr, female = (140 − age)*weight (kg)*0.85/72*Scr.

Abbreviations: A, aspirin; C, clopidogrel; Ccr, creatinine clearance rate; FBG, fasting blood glucose; HbA1c, glycated hemoglobin; HCY, homocysteine; HDL, high density lipoprotein; LDL, low density lipoprotein; Scr, serum creatinine; TC, total cholesterol; TG, triglycerides; UA, uric acid.

*p < 0.05.

### 3.5 Associated Factors of CMBs

In both univariable and multivariable logistic regression analyses, CMBs were independently associated with a history of HTN (unadjusted odds ratio [OR] = 2.092, 95% confidence interval [CI]: 1.087–4.023; adjusted odds ratio [AOR] = 2.071, 95% CI: 1.011–4.242) and with Fazekas Grade 3 WMH (OR = 4.073, 95% CI: 1.746–9.502; AOR = 4.376, 95% CI: 1.687–11.353) (Table 4).

**TABLE 4:** Independent predictors of microbleeds: logistic regression analysis.

Terms	Univariate		Multivariate	
	OR (95% CI)	*p* value	AOR (95% CI)	*p* value
Sex	1.180 (0.597–2.233)	0.64	1.162 (0.520–2.597)	0.71
Age	1.005 (0.982–1.028)	0.70	0.985 (0.955–1.016)	0.32
HTN	2.092 (1.087–4.023)	0.027*	2.071 (1.011–4.242)	0.047*
DM	0.967 (0.515–1.816)	0.92	0.861 (0.428–1.732)	0.68
Ccr	0.994 (0.981–1.008)	0.40	0.999 (0.983–1.015)	0.89
LDL	0.885 (0.641–1.222)	0.46	0.879 (0.618–1.250)	0.47
Fazekas score Grade 1	1.00 (ref)		1.00 (ref)	
Fazekas score Grade 2	1.455 (0.709–2.984)	0.31	1.561 (0.693–3.515)	0.28
Fazekas score Grade 3	4.073 (1.746–9.502)	0.001*	4.376 (1.687–11.353)	0.002*

Abbreviations: Ccr, creatinine clearance rate; DM, diabetes mellitus; HTN, hypertension; LDL, low density lipoprotein.

*p < 0.05.

## 4 Discussion

This study offers relevant insights into the prevalence, anatomical distribution patterns, and associated factors for CMBs in Chinese patients with AIS. The prevalence and risk factors for CMBs were examined among Chinese patients with MRI‐confirmed ischemic stroke, regardless of symptom severity or duration.

From a clinical perspective, CMBs represent more than incidental imaging findings; their burden may influence the risk–benefit assessment of secondary prevention strategies following AIS, particularly in the context of antiplatelet therapy, anticoagulation, and the risk of hemorrhage associated with intravenous thrombolysis and/or endovascular interventions. Accordingly, determining the population‐level prevalence of CMBs and identifying their correlates in Chinese patients with AIS may contribute to the development of imaging phenotype–informed, individualized treatment strategies.

Regarding antithrombotic medication, the prehospital use of dual antiplatelet therapy, single antiplatelet therapy, or anticoagulation was not found to be significantly associated with the presence, number, or anatomical distribution of CMBs. However, mixed‐type CMBs were slightly more prevalent among patients who had received dual antiplatelet therapy (10.8%) compared with those with deep/infratentorial (3.7%) or lobar (5.0%) CMBs. Although prior studies have suggested an association between aspirin or anticoagulant monotherapy and increased CMB burden, this discrepancy may be partially attributed to the exclusive inclusion of patients with AIS in the present cohort and the lack of analysis of the duration of antithrombotic therapy (Horstmann et al. 2015; Liu and Li 2015). CMB burden may have important clinical implications beyond its imaging value alone. A higher burden of CMBs may indicate increased hemorrhagic vulnerability and thus may be relevant when weighing the risks and benefits of antithrombotic therapy in patients with AIS. However, current evidence suggests that CMBs should not be interpreted in isolation or used as the sole basis for withholding antithrombotic treatment. Rather, CMB burden may serve as an imaging marker that contributes to individualized risk stratification in combination with stroke subtype, vascular risk profile, and the overall burden of cerebral small vessel disease (CSVD). Future prospective studies are needed to determine how CMB burden can be better integrated into treatment algorithms for antiplatelet and anticoagulant therapy (Zhao et al. 2024).

The observed prevalence of CMBs in this northern Chinese cohort was 49.1%, falling within the range previously reported in southern Chinese populations (27.8%–56.7%) (Soo et al. 2008; Fan et al. 2003; Zhou et al. 2010; Lei et al. 2018; Meng et al. 2020). These findings indicate a consistently higher prevalence of CMBs among Chinese patients when compared to Egyptian (26.3%), British (23%), and other Asian populations (19.5%) (Potigumjon et al. 2017; Nasreldein et al. 2025; Werring et al. 2005). This geographical variation may be attributed to differences in genetic susceptibility, vascular risk factor profiles, or neuroimaging protocols. Importantly, the detection of CMBs is highly sensitive to technical parameters of MRI. SWI typically identifies a greater number of microbleeds than T2*‐GRE sequences.

Factors such as magnetic field strength (1.5 T vs. 3.0 T), slice thickness, echo time, and post‐processing techniques can substantially influence detection rates. In the present study, a 3.0‐T MRI system with SWI was used, which has greater sensitivity for detecting microbleeds. As such, the prevalence observed may be higher than those reported in studies employing lower field strengths and/or T2*‐GRE sequences (Potigumjon et al. 2017; Nasreldein et al. 2025; Werring et al. 2005). The prevalence of CMBs in this cohort was higher than that reported in several southern Chinese cities (Soo et al. 2008; Fan et al. 2003; Zhou et al. 2010). However, one southern Chinese study reported an even higher prevalence, potentially due to its broader inclusion window of ≤ 30 days from symptom onset, in contrast to the ≤ 2‐week window applied in most other cohorts (Meng et al. 2020).

Lacunar infarction should be viewed as a distinct ischemic stroke subtype caused primarily by disease of the deep perforating arterioles, rather than simply a smaller form of other ischemic stroke mechanisms. Consistent with this concept, notable differences in CMB prevalence were observed across TOAST stroke subtypes, with the highest rates identified in patients with LAA (59.4%) and SVD (47.5%), and substantially lower rates in those with CE (21.4%) and ODC (25.0%). These findings are consistent with results from previous studies conducted in Egyptian and Thai populations, although other investigations have reported even higher CMB prevalence among patients with SVD (Potigumjon et al. 2017; Nasreldein et al. 2025; Kato et al. 2002; Ovbiagele et al. 2006). A significant association was observed between CMB burden and the proportion of patients with the SVD subtype, further supporting a shared microvascular etiology. Symptomatic lacunar stroke is also commonly accompanied by other neuroimaging markers of CSVD, particularly multiple CMBs and severe WMH, suggesting a diffuse underlying microangiopathy. The strong associations identified with both LAA and SVD subtypes support the hypothesis that overlapping pathological mechanisms may exist between LAA, microvascular aging, and the formation of CMBs (Csiszar et al. 2024). LAA and SVD may represent differing phenotypic manifestations along a continuum of chronic vascular injury, wherein age‐related atherosclerotic changes may extend to the cerebral microcirculation, promoting microvascular degeneration and contributing to CSVD‐related lesions, including CMBs (Csiszar et al. 2024; Ding et al. 2017). Prior studies have also shown that, compared with non‐lacunar ischemic stroke, lacunar stroke generally has a more favorable early functional prognosis, despite its important long‐term vascular implications. Taken together, these findings support the view that the pathophysiology, clinical features, imaging phenotype, and outcome of lacunar stroke are distinct from those of other acute ischemic cerebrovascular diseases (Arboix et al. 2010).

In contrast, CE represents a predominantly embolic pathophysiological mechanism and may be less closely linked to the cumulative structural injury of small cerebral vessels, which may explain the lower CMB burden observed in this subgroup when compared with LAA and SVD subtypes (Kim et al. 2016). The high proportion of mixed‐type CMBs observed in the cohort may reflect the coexistence of overlapping microangiopathies, including hypertensive arteriopathy and cerebral amyloid angiopathy (CAA), within the same patient (Ii et al. 2020).

Two independent factors were identified as being significantly associated with the presence of CMBs: HTN (AOR = 2.07) and severe WMH (AOR = 4.38), findings that are consistent with previous studies (Potigumjon et al. 2017; Nasreldein et al. 2025). The association between HTN and CMBs aligns with mechanistic evidence indicating that chronic HTN contributes to accelerated microvascular aging and increased vascular fragility, thereby predisposing patients to CMB formation (Csiszar et al. 2024). HTN has been consistently linked to both the presence and burden of CMBs in ischemic stroke cohorts, particularly in relation to deep and infratentorial CMBs, supporting hypertensive arteriopathy as a principal underlying mechanism (Agarwal et al. 2024; Sun et al. 2009; Klarenbeek et al. 2013).

The strong association observed between CMBs and severe WMH (Fazekas Grade 3) indicates a shared pathophysiological basis between these two manifestations of CSVD, potentially linked to common mechanisms such as HTN, which contributes to both microvascular leakage and white matter injury (Kato et al. 2002; Yamada et al. 2012; Kim and Lee 2013). The severity of WMH has been consistently associated with both the presence and burden of CMBs in acute stroke populations, supporting the interpretation that these lesions represent overlapping imaging markers of underlying CSVD (Duering et al. 2023; Wardlaw et al. 2013; Balestrieri et al. 2021; Liu et al. 2020; Fladt et al. 2018). Interestingly, lower levels of LDL cholesterol were observed in patients with a higher burden of CMBs, consistent with findings from a previous study indicating that reduced LDL cholesterol levels are associated with an increased risk of hemorrhagic transformation following acute ischemic cerebral infarction (Yang et al. 2016). In our cohort, the history of statin use did not differ significantly between groups, suggesting that the observed association is unlikely to be explained solely by statin use. Meta‐analytic data have proposed an inverse association between serum lipid levels and the presence of CMBs; however, residual confounding related to statin intensity, treatment duration, adherence, or other lipid‐lowering therapies cannot be excluded (Feng et al. 2021).

The observed distribution patterns of CMBs (23.8% strictly lobar, 32.1% strictly deep/infratentorial, and 44.1% mixed) indicate heterogeneity in the underlying pathogenic mechanisms. The proportion of patients with strictly lobar CMBs (23.8%) was comparable to that reported in Egyptian (20.5%) (Nasreldein et al. 2025) and African American (24%) populations (Shahjouei et al. 2017). A statistically significant association was found between TOAST stroke subtype and CMB location (*p* = 0.007). Lobar CMBs were most frequently observed among patients with LAA (80.0%), whereas SVD was more commonly represented in the deep/infratentorial (59.3%) and mixed (51.4%) CMB groups. These findings support the hypothesis that CMB distribution is not random but rather reflects distinct underlying vascular pathologies. Deep/infratentorial CMBs are classically associated with hypertensive arteriopathy affecting perforating arterioles, whereas strictly lobar CMBs may indicate the presence of CAA, particularly in older adults and in the presence of additional CAA‐related imaging markers (Klarenbeek et al. 2013; Wardlaw et al. 2013). However, in patients with AIS, the presence of lobar CMBs alone is insufficient to establish a diagnosis of definite CAA. In the absence of amyloid‐sensitive imaging or biomarkers, careful interpretation is required, and the application of formal diagnostic criteria including the Boston criteria version 2.0, may aid in etiologic classification (Klarenbeek et al. 2013).

The severity of WMH, graded by the Fazekas scale, also varied significantly according to CMB location phenotype (*p* = 0.048). The highest proportion of severe WMH (Grade 3) was observed in the mixed CMB group (45.9%), compared with the lobar (25.0%) and deep/infratentorial (25.9%) groups. This distribution pattern may indicate that mixed CMBs reflect a more advanced and widespread burden of CSVD, where multiple microangiopathic mechanisms coexist and jointly contribute to the formation of hemorrhagic lesions. Emerging literature supports the interpretation that mixed‐location CMBs often result from overlapping contributions of hypertensive arteriopathy and CAA, with composite imaging features capturing elements of both pathologies (Ii et al. 2020; Pasi et al. 2018).

### 4.1 Study Limitations

Several limitations should be considered. First, this was a retrospective single‐center study, and only patients who underwent SWI MRI were included. As a result, a substantial proportion of initially screened patients were excluded, which may have introduced selection bias and limited the generalizability of our findings. Second, the single‐center, retrospective design may limit the generalizability of the findings. Third, the absence of advanced amyloid‐sensitive imaging modalities restricted the ability to definitively differentiate between hypertensive arteriopathy and amyloid‐related microangiopathy. Four, the cross‐sectional study design precluded evaluation of temporal relationships between risk factors and the development of CMBs. Finally, the relatively small number of cases within certain stroke subtypes such as CE, may have reduced the statistical power to detect subtype‐specific associations. The limited representation of female patients further constrained the ability to perform adequately powered sex‐stratified analyses. Although sex was included as a covariate in the adjusted models, the potential for residual confounding and reduced precision in sex‐specific estimates cannot be excluded.

## 5 Conclusion

The findings of this study underscore the high prevalence and clinical relevance of CMBs in Chinese patients with AIS, particularly among those with LAA and SVD subtypes. HTN and severe WMH were associated with the presence of CMBs, while greater CMB burden was linked to a higher proportion of SVD and lower LDL cholesterol levels. In addition, patients with deep/infratentorial CMBs exhibited a higher prevalence of SVD and moderate‐to‐severe WMH compared with the strictly lobar CMB group. Further research is warranted to better understand the underlying mechanisms contributing to these associations. Prospective investigations incorporating longitudinal neuroimaging and advanced biomarkers, including amyloid positron emission tomography, may help define the temporal evolution and pathophysiological basis of CMBs across different stroke subtypes. In addition, integrating neuroimaging markers such as WMH, lacunes, and enlarged perivascular spaces into a comprehensive risk model may improve prediction of recurrent stroke, hemorrhagic events, and long‐term cognitive outcomes in patients with AIS. Future research should also focus on individualized decision‐making for intravenous thrombolysis, antithrombotic therapy, and post‐thrombectomy management, as well as on standardized MRI‐based and artificial intelligence‐assisted methods for the automated detection and quantitative assessment of CMBs.

## Author Contributions

**Ruiming Zhu**: data curation, formal analysis, software, funding acquisition, writing – original draft. **Xiangshu Cheng**: conceptualization, funding acquisition, writing – review and editing, project administration. **Mianwang He**: conceptualization, funding acquisition, writing – review and editing, project administration. **Yinglin Yang**: data curation, formal analysis, software, funding acquisition, writing – original draft. **Xiaoyan Chen**: data curation, software, formal analysis. **Lei Hou**: data curation, software, formal analysis.

## Funding

This work was supported by the National Key Research and Development Program of China (grant no. 2021YFC2701700 and 2021YFC2701703), the National Natural Science Foundation of China (82304739), the Henan Provincial Science and Technology Key Projects Program (252102311160), the Henan Provincial Medical Science and Technology Key Projects Program (SBGJ202402075), and the Henan Provincial Joint Construction Project of Medical Science and Technology Program (LHGJ20240393).

## Ethics Statement

This study was conducted with approval from the Ethical Committee of Chinese PLA General Hospital (approval number: S2025‐446‐01). This study was conducted in accordance with the Declaration of Helsinki. Written informed consent was obtained from all participants.

## Conflicts of Interest

The authors declare no conflicts of interest.

## Data Availability

All data generated or analyzed during this study are included in this article. Further enquiries can be directed to the corresponding author.

## References

[brb371495-bib-0001] Agarwal, A. , P. Ajmera , P. Sharma , and S. Kanekar . 2024. “Cerebral Microbleeds: Causes, Clinical Relevance, and Imaging Approach—A Narrative Review.” Journal of Neurosciences in Rural Practice 15, no. 2: 169–181. 10.25259/JNRP_351_2023. 38746527 PMC11090589

[brb371495-bib-0002] Arboix, A. , J. Massons , L. García‐Eroles , et al. 2010. “Nineteen‐Year Trends in Risk Factors, Clinical Characteristics and Prognosis in Lacunar Infarcts.” Neuroepidemiology 35, no. 3: 231–236. 10.1159/000319460. 20861654

[brb371495-bib-0003] Balestrieri, A. , P. Lucatelli , H. S. Suri , et al. 2021. “Volume of White Matter Hyperintensities, and Cerebral Micro‐Bleeds.” Journal of Stroke and Cerebrovascular Diseases 30, no. 8: 105905. 10.1016/j.jstrokecerebrovasdis.2021.105905. 34107418

[brb371495-bib-0004] Charidimou, A. , S. Shams , J. R. Romero , et al. 2018. “Clinical Significance of Cerebral Microbleeds on MRI: A Comprehensive Meta‐Analysis of Risk of Intracerebral Hemorrhage, Ischemic Stroke, Mortality, and Dementia in Cohort Studies (v1).” International Journal of Stroke 13, no. 5: 454–468. 10.1177/1747493017751931. 29338604 PMC6123529

[brb371495-bib-0005] Csiszar, A. , A. Ungvari , R. Patai , et al. 2024. “Atherosclerotic Burden and Cerebral Small Vessel Disease: Exploring the Link Through Microvascular Aging and Cerebral Microhemorrhages.” Geroscience 46, no. 5: 5103–5132. 10.1007/s11357-024-01139-7. 38639833 PMC11336042

[brb371495-bib-0006] Dar, N. Z. , Q. U. Ain , R. Nazir , and A. Ahmad . 2018. “Cerebral Microbleeds in an Acute Ischemic Stroke as a Predictor of Hemorrhagic Transformation.” Cureus 10, no. 9: e3308. 10.7759/cureus.3308. 32175198 PMC7053796

[brb371495-bib-0007] Ding, L. , Y. Hong , and B. Peng . 2017. “Association Between Large Artery Atherosclerosis and Cerebral Microbleeds: A Systematic Review and Meta‐Analysis.” Stroke and Vascular Neurology 2, no. 1: 7–14. 10.1136/svn-2016-000049. 28959485 PMC5435213

[brb371495-bib-0008] Duering, M. , G. J. Biessels , A. Brodtmann , et al. 2023. “Neuroimaging Standards for Research Into Small Vessel Disease‐Advances Since 2013.” Lancet Neurology 22, no. 7: 602–618. 10.1016/S1474-4422(23)00131-X. 37236211

[brb371495-bib-0009] Fan, Y. H. , L. Zhang , W. W. Lam , V. C. Mok , and K. S. Wong . 2003. “Cerebral Microbleeds as a Risk Factor for Subsequent Intracerebral Hemorrhages Among Patients With Acute Ischemic Stroke.” Stroke; A Journal of Cerebral Circulation 34, no. 10: 2459–2462. 10.1161/01.STR.0000090841.90286.81. 12958325

[brb371495-bib-0010] Feng, X. , Q. Tang , C. Cheng , and S. Xu . 2021. “Low Serum Lipid Levels, Use of Statin and Cerebral Microbleeds: A Systematic Review and Meta‐Analysis.” Journal of Clinical Neuroscience 94: 216–225. 10.1016/j.jocn.2021.10.032. 34863441

[brb371495-bib-0011] Fladt, J. , C. Kronlage , and G. M. De Marchis . 2018. “Cerebral White Matter Hyperintensities and Microbleeds in Acute Ischemic Stroke: Impact on Recanalization Therapies. A Review of the Literature.” Neuroscience Letters 687: 55–64. 10.1016/j.neulet.2018.09.003. 30194982

[brb371495-bib-0012] Greenberg, S. M. , M. W. Vernooij , C. Cordonnier , et al. 2009. “Cerebral Microbleeds: A Guide to Detection and Interpretation.” Lancet Neurology 8, no. 2: 165–174. 10.1016/S1474-4422(09)70013-4. 19161908 PMC3414436

[brb371495-bib-0013] Gregoire, S. M. , U. J. Chaudhary , M. M. Brown , et al. 2009. “The Microbleed Anatomical Rating Scale (MARS): Reliability of a Tool to Map Brain Microbleeds.” Neurology 73, no. 21: 1759–1766. 10.1212/WNL.0b013e3181c34a7d. 19933977

[brb371495-bib-0014] Horstmann, S. , M. Möhlenbruch , C. Wegele , et al. 2015. “Prevalence of Atrial Fibrillation and Association of Previous Antithrombotic Treatment in Patients With Cerebral Microbleeds.” European Journal of Neurology 22, no. 10: 1355–1362. 10.1111/ene.12608. 25557113

[brb371495-bib-0015] Ii, Y. , H. Ishikawa , H. Matsuyama , et al. 2020. “Hypertensive Arteriopathy and Cerebral Amyloid Angiopathy in Patients With Cognitive Decline and Mixed Cerebral Microbleeds.” Journal of Alzheimer's Disease 78, no. 4: 1765–1774. 10.3233/JAD-200992. PMC11062589 33185609

[brb371495-bib-0016] Kato, H. , M. Izumiyama , K. Izumiyama , A. Takahashi , and Y. Itoyama . 2002. “Silent Cerebral Microbleeds on T2*‐Weighted MRI: Correlation With Stroke Subtype, Stroke Recurrence, and Leukoaraiosis.” Stroke; A Journal of Cerebral Circulation 33, no. 6: 1536–1540. 10.1161/01.str.0000018012.65108.86. 12052987

[brb371495-bib-0017] Kim, B. J. , and S. H. Lee . 2013. “Cerebral Microbleeds: Their Associated Factors, Radiologic Findings, and Clinical Implications.” Journal of Stroke 15, no. 3: 153. 10.5853/jos.2013.15.3.153. 24396809 PMC3859003

[brb371495-bib-0018] Kim, B. J. , Y. Yoon , H. Sohn , D. W. Kang , J. S. Kim , and S. U. Kwon . 2016. “Difference in the Location and Risk Factors of Cerebral Microbleeds According to Ischemic Stroke Subtypes.” Journal of Stroke 18, no. 3: 297–303. 10.5853/jos.2016.00360. 27733027 PMC5066437

[brb371495-bib-0019] Klarenbeek, P. , R. J. van Oostenbrugge , R. P. Rouhl , I. L. Knottnerus , and J. Staals . 2013. “Higher Ambulatory Blood Pressure Relates to New Cerebral Microbleeds: 2‐Year Follow‐Up Study in Lacunar Stroke Patients.” Stroke; A Journal of Cerebral Circulation 44, no. 4: 978–983. 10.1161/STROKEAHA.111.676619. 23449261

[brb371495-bib-0020] Lei, C. , L. Zhong , Y. Ling , and T. Chen . 2018. “Blood Glucose Levels Are Associated With Cerebral Microbleeds in Patients With Acute Ischaemic Stroke.” European Neurology 80, no. 3–4: 187–192. 10.1159/000494990. 30572338

[brb371495-bib-0021] Liu, S. , and C. Li . 2015. “Antiplatelet Drug Use and Cerebral Microbleeds: A Meta‐Analysis of Published Studies.” Journal of Stroke and Cerebrovascular Diseases 24, no. 10: 2236–2244. 10.1016/j.jstrokecerebrovasdis.2015.05.022. 26272868

[brb371495-bib-0022] Liu, J. Y. , Y. J. Zhou , F. F. Zhai , et al. 2020. “Cerebral Microbleeds Are Associated With Loss of White Matter Integrity.” American Journal of Neuroradiology 41, no. 8: 1397–1404. 10.3174/ajnr.A6622. 32719091 PMC7658876

[brb371495-bib-0023] Meng, N. , W. Zhang , Y. Su , Z. Ye , and C. Qin . 2020. “Antiplatelet Therapy May be Safe in Ischemic Stroke Patients With Cerebral Microbleed.” Journal of International Medical Research 48, no. 8: 300060520949396. 10.1177/0300060520949396. 32814470 PMC7444122

[brb371495-bib-0024] Nagaraja, N. , N. Tasneem , A. Shaban , et al. 2018. “Cerebral Microbleeds Are an Independent Predictor of Hemorrhagic Transformation Following Intravenous Alteplase Administration in Acute Ischemic Stroke.” Journal of Stroke and Cerebrovascular Diseases 27, no. 5: 1403–1411. 10.1016/j.jstrokecerebrovasdis.2017.12.044. 29398533

[brb371495-bib-0025] Nasreldein, A. , A. Shoamnesh , N. Foli , et al. 2025. “Prevalence and Risk Factors of Cerebral Microbleeds Among Egyptian Patients With Acute Ischemic Stroke.” Neuroepidemiology 59, no. 3: 227–235. 10.1159/000540296. 39019020 PMC12136528

[brb371495-bib-0026] Ovbiagele, B. , J. L. Saver , N. Sanossian , et al. 2006. “Predictors of Cerebral Microbleeds in Acute Ischemic Stroke and TIA Patients.” Cerebrovascular Diseases 22, no. 5–6: 378–383. 10.1159/000094855. 16888379

[brb371495-bib-0027] Pasi, M. , A. Charidimou , G. Boulouis , et al. 2018. “Mixed‐Location Cerebral Hemorrhage/Microbleeds: Underlying Microangiopathy and Recurrence Risk.” Neurology 90, no. 2: e119–e126. 10.1212/WNL.0000000000004797. 29247070 PMC5772153

[brb371495-bib-0028] Potigumjon, A. , A. Watcharakorn , and P. A. Dharmasaroja . 2017. “Prevalence of Cerebral Microbleeds in Thai Patients With Ischemic Stroke.” Journal of Neurosciences in Rural Practice 8, no. 2: 216–220. 10.4103/0976-3147.203836. 28479795 PMC5402487

[brb371495-bib-0029] Shahjouei, S. , G. Tsivgoulis , M. Singh , et al. 2017. “Racial Difference in Cerebral Microbleed Burden Among Ischemic Stroke Patients.” Journal of Stroke and Cerebrovascular Diseases 26, no. 11: 2680–2685. 10.1016/j.jstrokecerebrovasdis.2017.06.040. 28838827

[brb371495-bib-0030] Soo, Y. O. , S. R. Yang , W. W. Lam , et al. 2008. “Risk vs Benefit of Anti‐Thrombotic Therapy in Ischaemic Stroke Patients With Cerebral Microbleeds.” Journal of Neurology 255, no. 11: 1679–1686. 10.1007/s00415-008-0967-7. 19156486

[brb371495-bib-0031] Sun, J. , Y. O. Soo , W. W. Lam , K. S. Wong , J. S. Zeng , and Y. H. Fan . 2009. “Different Distribution Patterns of Cerebral Microbleeds in Acute Ischemic Stroke Patients With and Without Hypertension.” European Neurology 62, no. 5: 298–303. 10.1159/000235850. 19729926

[brb371495-bib-0032] Wahlund, L. O. , F. Barkhof , F. Fazekas , et al. 2001. “A New Rating Scale for Age‐Related White Matter Changes Applicable to MRI and CT.” Stroke; A Journal of Cerebral Circulation 32, no. 6: 1318–1322. 10.1161/01.str.32.6.1318. 11387493

[brb371495-bib-0033] Wardlaw, J. M. , E. E. Smith , G. J. Biessels , et al. 2013. “Neuroimaging Standards for Research Into Small Vessel Disease and Its Contribution to Ageing and Neurodegeneration.” Lancet Neurology 12, no. 8: 822–838. 10.1016/S1474-4422(13)70124-8. 23867200 PMC3714437

[brb371495-bib-0034] Werring, D. J. , L. J. Coward , N. A. Losseff , H. R. Jäger , and M. M. Brown . 2005. “Cerebral Microbleeds Are Common in Ischemic Stroke but Rare in TIA.” Neurology 65, no. 12: 1914–1918. 10.1212/01.wnl.0000188874.48592.f7. 16380612

[brb371495-bib-0035] Yamada, S. , M. Saiki , T. Satow , et al. 2012. “Periventricular and Deep White Matter Leukoaraiosis Have a Closer Association With Cerebral Microbleeds Than Age.” European Journal of Neurology 19, no. 1: 98–104. 10.1111/j.1468-1331.2011.03451.x. 21645176

[brb371495-bib-0036] Yang, N. , M. Lin , B. G. Wang , et al. 2016. “Low Level of Low‐Density Lipoprotein Cholesterol Is Related With Increased Hemorrhagic Transformation After Acute Ischemic Cerebral Infarction.” European Review for Medical and Pharmacological Sciences 20, no. 4: 673–678. 26957269

[brb371495-bib-0037] Zhao, B. , Y. Yuan , Z. Li , et al. 2024. “Risk of Intracranial Hemorrhage in Patients Using Anticoagulant Therapy for Atrial Fibrillation After Cerebral Microbleeds Combined With Acute Ischemic Stroke: A Meta‐Analysis.” Frontiers in Neurology 15: 1372231. 10.3389/fneur.2024.1372231. 38560733 PMC10978779

[brb371495-bib-0038] Zhou, Z. M. , S. Yang , X. Y. Yue , et al. 2010. “[Influencing Factors for Cerebral Microbleeds in Patients With Acute Ischemic Stroke].” [In Chinese.] Zhonghua Yi Xue Za Zhi 90, no. 7: 451–453. 20368066

